# Divergent cerebrospinal fluid cytokine network induced by non-viral and different viral infections on the central nervous system

**DOI:** 10.1186/s12879-015-1035-4

**Published:** 2015-08-19

**Authors:** Michele Souza Bastos, Jordana Grazziela Coelho-dos-Reis, Danielle Alves Gomes Zauli, Felipe Gomes Naveca, Rossicleia Lins Monte, João Paulo Pimentel, Valéria Munique Kramer Macário, Natália Lessa da Silva, Vanessa Peruhype-Magalhães, Marcelo Antônio Pascoal-Xavier, Allyson Guimaraes, Andréa Teixeira Carvalho, Adriana Malheiro, Olindo Assis Martins-Filho, Maria Paula Gomes Mourão

**Affiliations:** Tropical Medicine Foundation Dr. Heitor Vieira Dourado, Manaus, AM Brazil; Amazonas State University, Manaus, AM Brazil; Laboratory of Biomarkers for Diagnosis and Monitoring, René Rachou Research Center, FIOCRUZ, Av. Augusto de Lima 1715, Barro Preto, Belo Horizonte, Minas Gerais CEP 30190-002 Brazil; Institute Leônidas and Maria Deane – Fiocruz Amazônia, Manaus, AM Brazil; Hematology and Hemotherapic Foundation of Amazonas, Manaus, AM Brazil; Department of Anatomic Pathology, Medicine School, Federal University of Minas Gerais, Belo Horizonte, MG Brazil; Instituto Hermes Pardini, Belo Horizonte, MG Brazil

## Abstract

**Background:**

Meningoencephalitis is one of the most common disorders of the central nervous system (CNS) worldwide. Viral meningoencephalitis differs from bacterial meningitis in several aspects. In some developing countries, bacterial meningitis has appropriate clinical management and chemotherapy is available. Virus-associated and virus not detected meningoencephalitis are treatable, however, they may cause death in a few cases. The knowledge of how mediators of inflammation can induce disease would contribute for the design of affordable therapeutic strategies, as well as to the diagnosis of virus not detected and viral meningoencephalitis. Cytokine-induced inflammation to CNS requires several factors that are not fully understood yet.

**Methods:**

Considering this, several cytokines were measured in the cerebrospinal fluid (CSF) of patients with undiagnosed and viral meningoencephalitis, and these were correlated with cellularity in the CSF.

**Results:**

The results demonstrate that an altered biochemical profile alongside increased cellularity in the cerebrospinal fluid is a feature of patients with meningoencephalitis that are not associated with the detection of virus in the CNS (*P* < 0.05). Moreover, HIV-positive patients (*n* = 10) that evolve with meningoencephalitis display a distinct biochemical/cytological profile (*P* < 0.05) in the cerebrospinal fluid. Meningoencephalitis brings about a prominent intrathecal cytokine storm regardless of the detection of virus as presumable etiological agent. In the case of Enterovirus infection (*n* = 13), meningoencephalitis elicits robust intrathecal pro-inflammatory cytokine pattern and elevated cellularity when compared to herpesvirus (*n* = 15) and Arbovirus (*n* = 5) viral infections (*P* < 0.05).

**Conclusion:**

Differences in the cytokine profile of the CSF may be unique if distinct, viral or presumably non-viral pathways initially trigger the inflammatory response in the CNS.

## Background

Meningoencephalitis is one of the most common disorders of the central nervous system (CNS) in children worldwide [1]. Viral meningoencephalitis differs from bacterial meningitis, which has well-established clinical management and chemotherapy is available. Most cases of meningoencephalitis are of viral aetiology such as: Epstein-Barr virus, herpes simplex viruses, varicella-zoster virus, Influenza virus, Arboviruses, lymphocytic choriomeningitis virus, mumps virus, measles virus and human enteroviruses. Human enteroviruses are the most common causes of viral meningoencephalitis [2–6]. However, some meningoencephalitis patients may test negative for any virus known and are, therefore, considered, as virus not detected meningoencephalitis.

Even after the wide introduction of vaccine for some of those viruses (eg: mumps), viral meningitis still is a major public health issue in several developing and developed countries. Although meningoencephalitis is usually a benign disease and recovery may occur within 7 to 10 days, it may exceptionally lead to serious complications or death. There is no specific treatment for meningoencephalitis, but in some instances specific treatment is available depending on the virus (such as herpes virus) [1]. Convalescence from meningoencephalitis is thus relies almost entirely upon the immune system and its role on clearing viral infection and/or regulating the inflammatory response in the meninges.

Cytokine-induced inflammation to CNS requires several factors that will lead ultimately to the blood-brain barrier (BBB) disruption. BBB breakdown renders the CNS open to several systemic factors such as the inflammatory mediators associated to the meningoencephalitis and meningitis [7]. Among those mediators, recruitment of both innate and adaptive immune cells such as neutrophils, lymphocytes and monocytes are important to the increased cellularity and local inflammation. In this regard, levels of interleukin-6 (IL-6) and tumor necrosis factor alpha (TNF-α) were measured in cerebrospinal fluids from patients with meningoencephalitis. IL-6 was increased in aseptic and bacterial meningitis, whereas TNF-α was increased only in bacterial [8–10].

Intrathecal production of IL-10 seems alleviate pain and modulate neuroinflammation in the CNS mediated by resident leukocytes [11–14]. Considering these findings, measuring CSF inflammatory cytokine levels in patients with acute meningitis and meningoencephalitis could be a valuable diagnostic tool. Using these molecules as biomarkers of disease morbidity could improve the prognosis of patients with meningoencephalitis, allowing for a more rapid and accurate follow-up of these patients [8, 9]. These biomarkers could be also important targets for future therapeutic approaches to the neglected infectious or undiagnosed meningoencephalitis [11–14].

## Methods

### Study population

This study was designed as a cross-sectional study of the cerebrospinal fluid from patients with ascetic meningitis at the public health hospital for Infectious Diseases in Manaus (FMT-HVD). The samples were selected according to the eligibility criteria and the study had approval by the Ethics Committee from the FMT-HVD. All patients signed an informed written consent in accordance to the guidelines established by the 466/2012 resolution at the Brazilian National Health Council.

A total of 165 patients with suspected CNS infection were screened from January 2010 through August 2012. One hundred and twenty-nine patients came from the public health hospital for Infectious Diseases in Manaus and the other 36 CSF samples were from different hospitals of the city. Symptomatic infection of the CNS was confirmed by the presence of acute neurological signs and symptoms such as headache, fever, focal neurologic findings and altered consciousness. Lumbar puncture was performed in all the patients on the day of hospital admission. An aliquot of the CSF was submitted for routine analysis: total and differential cells count (>5 cells/mm3); determination of protein, lactate and glucose, and microbiological tests for bacteria and fungi. Patients with confirmed bacterial and fungal infections were excluded from the study. Patients with asseptic menigitis with or without viral infections were included in the study.

### Diagnosis of viral infection in the CSF

Diagnosis of specific virus infection of CSF was determined for several virus. For that, both DNA and RNA were extracted from 200 μl and 140 μl of CSF samples respectively using the QIAamp Viral DNA and RNA Mini Kit (QIAGEN, USA). For the detection of five herpesvirus: herpes simplex virus type 1and 2 (HSV-1 and HSV-2), Cytomegalovirus (CMV), varicella zoster virus (VZV), Epstein-Barr virus (EBV) in CSF samples, the protocol (multiplex PCR) described elsewhere was applied [15]. For detection of flaviviruses (DENV-1, DENV-2, DENV-3, DENV-4, yellow fever virus (YFV), Rocio virus (ROCV), Ilheus virus (ILHV) and SLEV and alphaviruses (Western equine encephalitis virus (WEEV), Eastern equine encephalitis (EEEV), Venezuelan equine encephalitis virus (VEEV) and Mayaro virus (MAYV)), the methods described by Bronzoni et al. were used [16, 17]. The detection of Orthobunyavirus Oropouche was performed as described elsewhere [18]. The detection of the Enterovirus genus (EV) was performed as described elsewhere [19]. To assure success of RNA purification and RT-PCR, GAPDH was amplified. For DNA purification, β-actin was used as housekeeping gene for PCR control.

### Flow cytometric quantitative analysis of cytokines in the Cerebrospinal Fluid

The cytokine levels of IL-6, IL-10, IL-12, TNF-α, IFN-γ and IL-17A were measured in Cerebrospinal fluid (CSF) from all patients by Cytometric Bead Array (BD™ HU T_H_1, T_H_2, T_H_17 CBA Kit - code 560484, BD® Biosciences, San Jose, CA, USA). The CSF cytokine levels were measured as per manufacturer’s instructions and modified as previously described [20]. Data acquisition was performed by flow cytometry using a FACSCalibur flow cytometer (Becton Dickinson, La Jolla, CA, USA). The results were expressed as pg/mL, as assessed by the standard curve.

### Statistical and network analysis

To model the complex interactions between the biomarkers evaluated in the study, networks were assembled by, first, assessing the association within the cytokines secreted in CSF for each clinical group. Spearman’s correlation test was performed to assess the association between the levels of each cytokine (pg/mL) and the percentage of cell subsets tested. The level of cytokines measured in the CSF obtained from patients with meningoencephalitis were compared by non-parametric tests based on analysis of variance (anova), Kruskal–Wallis test followed by Dunn’s multiple comparison test. The Graphpad Prism software version 5.0 (San Diego, CA, USA) was used for data analysis and the Cytoscape software version 3.0.1 (Cytoscape Consortium San Diego, CA, USA) was employed for computing the subsystems composed of networks of the biomolecules [21].

## Results

### Altered biochemical parameters alongside increased cellularity in the cerebrospinal fluid is a feature of patients with meningoencephalitis and with no association with the presence of virus in the CNS

**Fig. 1:**
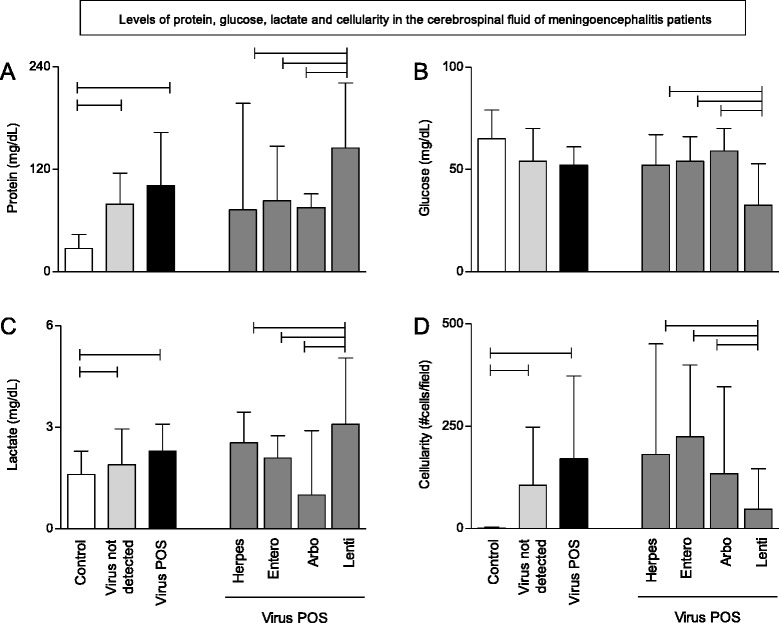
Biochemical profile of cerebrospinal fluid (CSF) in meningoencephalitis patients. Levels of protein (**a**) glucose (**b**), lactate (**c**), and cellularity (**d**) were measured in the CSF of control samples (white), virus not detected or undiagnosed (light gray) and virus-positive (black) patients with meningoencephalitis as indicated in material and methods. Virus-positive patients were subdivided according to the type of viral infection: Herpersvirus, Enterovirus, Arbovirus and Lentivirus (dark gray). Results are expressed as bars that represent median values with interquartile range as error bars. Horizontal connecting lines represent statistical differences between groups when *p* < 0.05

In order to understand the biochemical profile of patients with meningoencephalitis with virus-positive (*n* = 43) or undiagnosed (virus not detected) (*n* = 80) patients, the levels of protein, glucose and lactate along with cellularity were tested in the cerebrospinal fluid samples of those patients and healthy controls. Cellularity above 5 cells/field was considered as positive. Results are presented in Fig. 1. The results indicate that despite increased protein and lactate levels alongside influx of mononuclear cells to the cerebrospinal fluid of patients with meningoencephalitis, these altered parameters are unable to distinguish statistically patients with or without viral infection.

### HIV-positive patients that evolve with meningoencephalitis display a distinct biochemical/ cytological profile in the cerebrospinal fluid

Meningoencephalitis patients with viral infections were subdivided by the viral family - Herpesvirus (Herpes; *n* = 15), Enterovirus (Entero; *n* = 13), Arbovirus (Arbo; *n* = 5) and Lentivirus (Lenti; *n* = 10) and their biochemical profile was contrasted. The results (Fig. 1) indicate that the Lenti group presented statistically increased protein and lactate levels when compared to the other virus (+) groups, whereas the former showed statistically decreased glucose levels and cellularity. The HIV-positive group (Lenti) clearly displayed a distinct biochemical/cytological profile in the cerebrospinal fluid when compared to Meningoencephalitis patients infected with Herpesvirus, Enterovirus and Arbovirus, as evidenced by their median and interquartile range values.

### Meningoencephalitis brings about a prominent intrathecal cytokine storm regardless of the presence of virus as presumable etiological agent

**Fig. 2:**
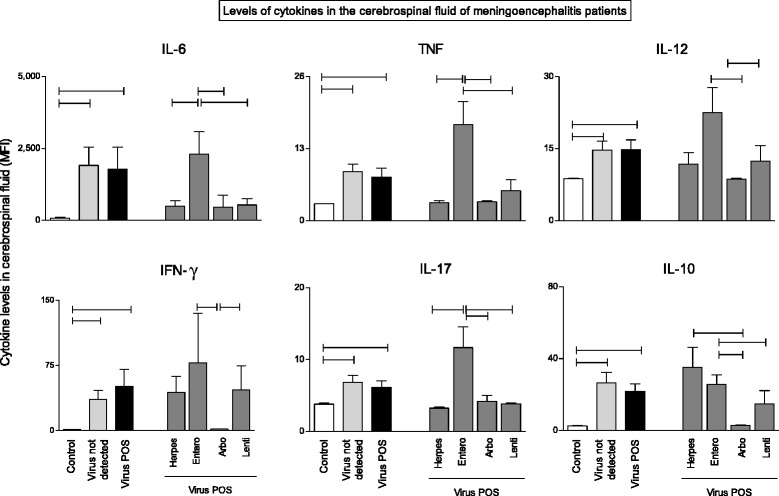
Cytokine profile of cerebrospinal fluid (CSF) in meningoencephalitis. Levels of the cytokines IL-6, IL-12, IL-17, TNF-α, IFN-γ and IL-10 were measured in the CSF of control samples, virus not detected (undiagnosed) and virus-positive patients with meningoencephalitis. Cytokine levels in the CSF were evaluated by cytometric beads array (CBA) as described in Material and Methods. Results are expressed as Mean Fluorescence Intensity (MFI) plotted in bars with standard error. Statistical differences between groups are highlighted as connecting lines

The cytokines IL-6, IL-10, IL-12, IL-17, TNF-α and IFN-γ were evaluated in the Cerebrospinal fluid (CSF) of patients with meningoencephalitis with and without viral infections as well as in control CSF samples using the cytometric bead array (CBA – BD biosciences). Results are illustrated in Fig. 2. The levels of all cytokines tested were significantly higher in patients with meningoencephalitis regardless of the presence of viral infection. Meningoencephalitis presumably caused by Enterovirus infection elicits robust intrathecal pro-inflammatory cytokine pattern, with statistically higher levels of IL-6, TNF and IL-17. The inverse is observed for Arbovirus infection. Enterovirus and Lentivirus groups showed significantly higher levels of IL-12 and IFN-γ when compared to Arbovirus group. Enterovirus and Herpesvirus groups showed significantly higher levels of IL-10 when compared to Arbovirus group (Fig. 2).

### High cerebrospinal fluid cellularity is associated with vigorous pro-inflammatory intrathecal cytokine profile regardless the presence of CNS viral infection

**Fig. 3:**
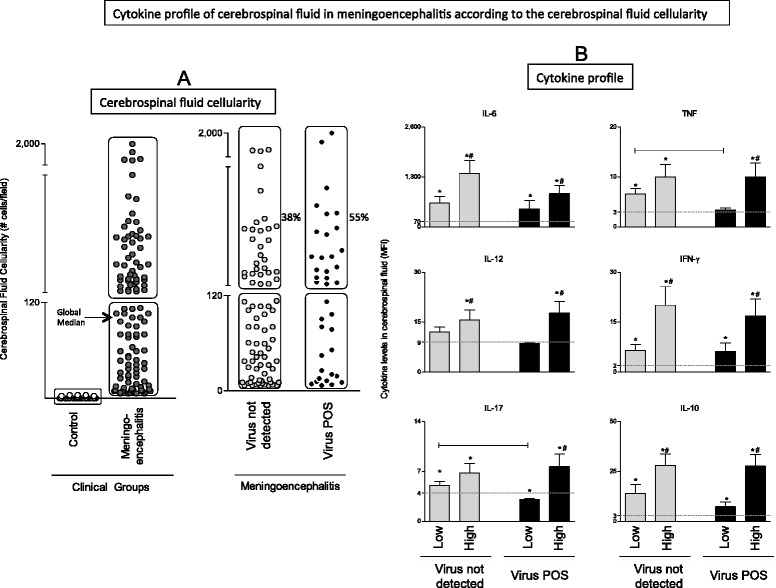
Cerebrospinal fluid cellularity was measured in control samples, virus not detected (undiagnosed) and virus-positive patients with meningoencephalitis (**a**) as described in material and methods. Results are expressed as number of cells per field (#cells/field). The global median of meningoencephalitis group was calculated and employed as a cut off point to discriminate patients with low and high cellularity. Percentages of high cellularity are displayed for virus not detected (undiagnosed) and virus-positive patients with aseptic meningitis. **b** Virus not detected (undiagnosed) and virus-positive patients with meningoencephalitis were classified as with either high or low cellularity and subdivided in two groups. The levels of the cytokines IL-6, IL-12, IL-17, TNF-α, IFN-γ and IL-10 (expressed as Mean Fluorescence Intensity-MFI) of the subgroups were compared. Statistical differences between subgroups within the same group are highlighted as connecting lines or # and differences between groups (black versus gray) were highlighted as *. The median values for each cytokine measured in the control sample are represented by a dotted line in each graph

The cellularity in the CSF was evaluated in patients with meningoencephalitis and healthy controls (Fig. 3). All patients with meningoencephalitis had cellularity above this threshold and controls had cellularity below it. Using the total number of samples, a global median was calculated in order to establish an internal cut off point to differentiate low from high cellularity. Patients with viral meningoencephalitis displayed higher frequency of high cellularity (55 %) when compared with meningoencephalitis patients without viral infection (38 %), however this difference was not statistically significant (Fig. 3a).

Subsequently, patients with high and low cellularity were segregated in separate groups and their cytokine levels were contrasted. Patients with high cellularity present elevated levels of cytokines regardless of viral infection except for the levels of IL-6. Both groups composed of high and low cellularity displayed similar levels of IL-6 in the CSF. When the cytokine levels of patients with low cellularity were contrasted, patients with undiagnosed meningoencephalitis presented levels of TNF-α and IL17 statistically higher than patients with virus-positive meningoencephalitis (Fig. 3b).

### Meningoencephalitis evolving with low cellularity is associated with augmented levels of TNF and IL-17 particularly in the absence of presumed viral etiology

**Fig. 4:**
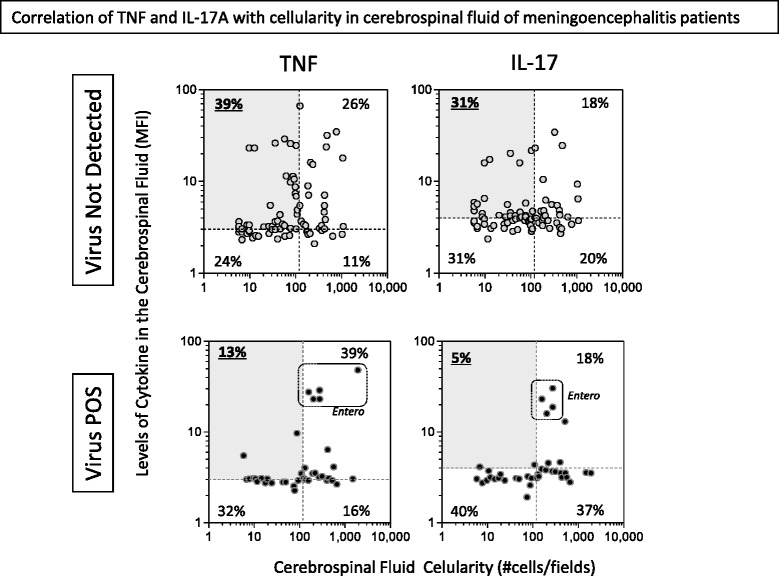
Association of the cytokine profile of cerebrospinal fluid with cellularity in aseptic meningitis. The levels of TNF-α and IL-17 as well as cellularity were measured in the CSF as described in material and methods. Graphs of cellularity (x axis) versus cytokine (y axis) were plotted and each dot represents one patient from (top panels) virus not detected (undiagnosed) and (bottom panels) virus-positive meningoencephalitis groups. Dotted lines represent the global median of cellularity (vertical) and cytokine (horizontal) and the percentages express the frequency of patients in each quadrant

Previously, the results demonstrated that TNF-α and IL17 were secreted in different magnitude in groups with high and low cellularity. Therefore, further analyses of these two cytokines were performed. First, the level of the cytokines and cellularity were correlated by plotting a “cellularity-versus-cytokine” dual-axis graph. The thresholds in the graph represent the global median values (horizontal = cellularity; Transversal = cytokine), which formed a 4-quarter dot-plot. Figure 4 displays the results for TNF-α and IL17 plots. The frequency of patients with viral meningoencephalitis (TNF-α and IL-17 = 55 %) was higher in the high cellularity quarters (top and bottom right) of the 4-quarter graph when compared to frequency of patients with undiagnosed meningoencephalitis in those quarters (TNF-α and IL-17 = 37–38 %). In addition, undiagnosed meningoencephalitis patients had higher values of the upper-left corner of the graph (high cytokine levels with low cellularity) for both TNF-α (39 %) and IL-17 (31 %) when compared to patients with virus-positive meningoencephalitis (TNF-α = 13 % and IL-17 = 5 %). Patients from the Enterovirus group are observed in the upper right corner of both graphs confirming that this group presents high cytokine production accompanied by greater cellularity.

### Meningoencephalitis evolving with low cellularity in the absence of presumed viral etiology shows an imbricate cytokine network with a strong TNF/IL-17 axis

**Fig. 5:**
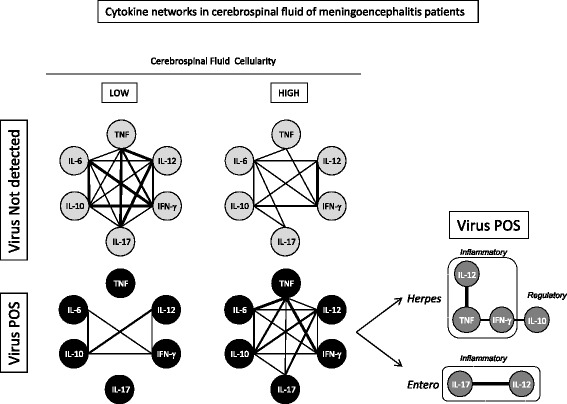
CSF cytokine networks in meningoencephalitis patients with high and low cellularity. Association among cytokines is displayed as interactive nets for virus not detected (gray) and virus-positive (black) meningoencephalitis patients. Each connection corresponds to statistical positive correlation between the cytokine pairs (*p* < 0.05)

Considering the intertwined collaboration between cellularity and cytokines and among the cytokines to produce the inflammatory response in meningoencephalitis, an interactive analysis was performed, seeking for correlations between the biomarkers tested. Networks of cytokines in high and low cellularity groups were built and are displayed in Fig. 5. Results in gray circle show data regarding undiagnosed meningoencephalitis, whereas results in black rectangles represent virus-positive meningitis.

A highly connected net was found in the group of undiagnosed meningitis with low cellularity and again in the group of virus-positive meningoencephalitis with high cellularity. The diad TNF-α/IL-17 is seen but exclusively in the network composed of data from undiagnosed meningoencephalitis patients with low cellularity. The diads IL-10/IFN-γ and IL-6/IL-17 are present only in undiagnosed meningoencephalitis patients regardless of cellularity. The interactive networks display a unique view of the CSF compartment, composed of several interactions. These interactions may possibly indicate that the relationship of cytokines may be associated with the nature or cause of the inflammatory response, which is characterized as active and abundant in connections in the undiagnosed low cellularity meningoencephalitis and again in the virus-positive high cellularity meningoencephalitis. During these two clinical situations, the cytokines seem to communicate and contribute to the process of inflammation in meningoencephalitis. In regards to the association with viral infection, Herpesvirus-associated meningoencephalitis displays an IL-10-modulated inflammatory profile, whereas Enterovirus is especially characterized by the IL-17/IL-12 diad inflammatory profile.

## Discussion

The present study sheds light on the interactions between cytokines in the CSF on virus negative and positive meningoencephalitis. These interactions may be associated with the origins of inflammatory response, which is characterized as active and abundant in connections in the undiagnosed low cellularity meningoencephalitis and again in the virus-positive high cellularity meningoencephalitis. During these two clinical situations, the cytokines seem to communicate and contribute to the process of inflammation in meningoencephalitis.

Strategies for therapeutic approaches in the context of virus-associated and undiagnosed meningoencephalitis are unavailable and in a few cases may cause death. The knowledge of how mediators of inflammation can induce disease would contribute for the design of novel therapeutic strategies, as well as to the diagnosis and prognosis of undiagnosed and viral meningoencephalitis. Cytokine-induced inflammation to CNS encompasses several factors that are yet to be fully understood. Considering this, several cytokines were measured in the CSF of patients with undiagnosed and viral meningoencephalitis, and these were correlated with cellularity in the CSF.

Previous reports have identified a prominent secretion of pro-inflammatory cytokines in the CNS. Pro-inflammatory chemokine CXCL8 was increased in viral meningitis, whereas IFN-γ was increased only in the meningitis cause by mumps and enteroviral infection [22]. Levels of IL-6 seem to be increased in meningitis regardless of the presence and type of infection (bacteria or virus), making this cytokine an ideal biomarker for meningoencephalitis prognosis [8, 10, 23]. Meningoencephalitis composed of high and low cellularity displayed similar levels of IL-6 in the CSF, indicating that the secretion of this cytokine is probably independent of the cell influx to the CNS.

Regarding the TNF-α, previous reports suggest that TNF-α levels are increased only in bacterial meningitis [10]. The present results challenge this finding, since increased levels of TNF-α were found in the CSF of patients with meningoencephalitis. TNF-α was found as an important biomarker for identifying patients with undiagnosed meningoencephalitis with low cellularity, which is in agreement with recent reports that recommend the evaluation of TNF-α levels in the diagnosis of bacterial and meningoencephalitis [24].

The appearance of leucocytes in the CSF is a hallmark of meningoencephalitis regardless of the aetiological agent. Previous study revealed that distinct viruses may cause different responses in the activity of gelatinases, enzymes that are crucially involved in leucocyte trafficking within the CNS [25, 26]. Increase in adhesion molecules in cerebrospinal fluid was observed in children with mumps meningitis, which may be associated with the intense cell influx into the CNS [26]. Cytokines are also crucial candidate mediators of cell migration from blood into the CNS. Cell migration into the CNS has already been associated with increased levels of IL-1, IL-1R2, IL-6; IL-8, TNF-α and IFN-γ in the CSF of children with mumps meningitis, enteroviral meningitis and without CNS infection [10, 22, 23].

Understanding the Th1/Th2 cytokine balance in cerebrospinal fluid of patients with meningoencephalitis may also shed light into the different pathogenic process and local inflammatory response in the CNS during aseptic meningitis. In this regard, several ratios between cytokines were altered such as IFN-γ/IL-10, IFN-γ/TNF-α and IFN-γ/IL-17 (data not shown) in patients with meningoencephalitis. Those ratios were significantly higher in virus positive when compared to virus negative-meningoencephalitis patients, indicating a predominant Th1 response in virus positive-meningoencephalitis and a predominant Th2 response in undiagnosed meningoencephalitis (data not shown).

On the other hand, TNF-α/IL-10 and IL-17/IL-10 ratios were significantly lower in virus positive when compared to undiagnosed meningoencephalitis patients. These ratios may indicate poor prognosis due to lower production of IL-10. There is clinical and experimental evidence that this modulatory cytokine has shown a therapeutic potential for treatment of CNS-related illnesses [11, 12]. Recent findings have demonstrated that release of plasmid DNA-encoding IL-10 from PLGA microparticles facilitates long-term reversal of neuropathic pain following a single intrathecal administration [11]. In addition, there is experimental evidence that upregulation of IL-10 expression in the CSF suppresses neuroinflammation in mouse model [12–14].

The results demonstrate a unique and abundant cytokine profile of cerebrospinal fluid in meningoencephalitis IL-17 levels were statistically higher in patients with meningoencephalitis without viral infection as compared to those with viral infections. IL-17 seem to play an important role in lesions associated to the autonomic and central nervous system such as multiple sclerosis. Activation of the IL-17/IL-8 axis in the CSF induces heavy neutrophil infiltration and contributes to extensive spinal cord lesion formation in multiple sclerosis [27]. These results are in agreement with the present findings that indicate that patients with undiagnosed meningoencephalitis presented levels of IL-17 and TNF-α statistically higher than patients with virus-positive meningitis in the presence of low cellularity.

At last, network analysis revealed that the diad TNF-α/IL-17 axis is seen exclusively in the network composed of data from undiagnosed meningoencephalitis patients with low cellularity. Corroborating these results, recent study has shown that increased CSF levels of IFN-γ and IL-17A in syphilitic patients with CSF abnormalities suggest that cells of adaptive immunity (probably T-helper cells producing IFN-γ and IL-17) may contribute to the inflammatory response present in the CNS in the neurosyphilis. In addition, the lack of correlation between serum and CSF IL-17A levels in previous findings suggests intrathecal production of this cytokine [28].

Differences in the cytokine profile may be unique if distinct pathways initially trigger the inflammatory response. Enterovirus and Lentivirus groups showed significantly higher levels of IL-12 and IFN-γ, demonstrating a pro-inflammatory response in the onset of those viral infections in the CNS. Arbovirus group presented significantly lower cytokine levels in the CSF, when compared to other virus groups, which could indicate that this virus could be hidden from the immune system. More detailed studies should be performed in order to elucidate the mechanisms involved in the processes regarding the *de novo* production and secretion of cytokines and their role during viral infection on the CNS. All in all, these results indicate that the prominent cytokine secretion alongside higher influx of mononuclear cells to the CSF is associated with meningoencephalitis regardless of the presence of virus. It is important to conjecture that the possible presence of viruses that were not tested in samples, which were assigned as undiagnosed meningoncephalitis cases could affect the outcomes of the study to a certain extent, and this should be considered as a possible limitation of the study.

## Conclusions

Differences in the cytokine profile of the CSF may be unique if distinct pathways initially trigger the inflammatory response in the CNS.

## Abbreviations

Arbo: Arbovirus group
BBB: Blood brain barrier
CNS: Central Nervous System
CSF: Cerebrospinal fluid
CMV: Cytomegalovirus
DENV: Dengue virus
EBV: Epstein-Barr virus
EEEV: Eastern equine encephalitis
Entero: Enterovirus group
EV: Enterovirus
HIV: Human Immunodeficiency virus
Herpes: Herpesvirus group
HSV-1/2: Herpes simplex virus type 1/2
VZV: Varicella zoster virus
IFN-γ: Interferon-gamma
IL-6: Interleukin-6
IL-10: Interleukin-10
IL-12: Interleukin-12
IL-17A: Interleukin-17A
ILHV: Ilheus virus
Lenti: Lentivirus group
FMT-HVD: Public health hospital for Infectious Diseases in Manaus
FAYV: Mayaro virus
TNF-α: Tumor Necrosis Factor alpha
VEEV: Venezuelan equine encephalitis virus
WEEV: Western equine encephalitis virus
YFV: Yellow Fever Virus
